# Longitudinal Trajectories of Albuminuria and eGFR in Type 2 Diabetes Mellitus: Natural Progression of Diabetic Kidney Disease

**DOI:** 10.1155/jdr/9269085

**Published:** 2025-08-18

**Authors:** Xiuqi Qiao, Qingyun Cai, Yanxiang Luo, Lixin Guo, Qi Pan

**Affiliations:** ^1^Department of Endocrinology, Beijing Hospital, National Center of Gerontology, Institute of Geriatric Medicine, Chinese Academy of Medical Sciences, Beijing, China; ^2^Graduate School of Peking Union Medical College, Chinese Academy of Medical Sciences, Beijing, China; ^3^Fifth School of Clinical Medicine, Peking University, Beijing, China

**Keywords:** albuminuria, diabetic kidney disease, temporal trends, Type 2 diabetes mellitus

## Abstract

**Background:** This longitudinal cohort study is aimed at examining the natural progression trajectories of diabetic kidney disease (DKD) in Type 2 diabetes mellitus (T2DM), assessing estimated glomerular filtration rate (eGFR) and albuminuria patterns over time.

**Methods:** This longitudinal observational study analyzed 694 hospitalized patients with T2DM, featuring a cohort with a median age of 59.0 years (interquartile range [IQR] 52.2–67.0) and a median diabetes duration of 12.0 years (IQR 6.0–18.0). The baseline population included 258 female participants (37.2%), with longitudinal data collected over a 4.5-year follow-up period conducted between March 2013 and January 2025. DKD was diagnosed per the 2024 ADA Standards of Medical Care in Diabetes. Disease severity stratification employed two validated biomarkers: urinary albumin excretion rate (AER) and eGFR calculated using the Chronic Kidney Disease Epidemiology Collaboration equation.

**Results:** The analytical cohort comprised 421 participants in the urinary AER group and 487 in the eGFR group. Baseline albuminuria stratification revealed 61.3% normoalbuminuria, 36.6% microalbuminuria, and 2.4% macroalbuminuria. Over 4.5 years of follow-up, 9.9% demonstrated albuminuria stage progression. The prevalence of G1, G2, G3, and G4 was 51.4%, 37.7%, 10.5%, and 0.5%, respectively, with 32.5% exhibiting renal function deterioration. Albuminuria progression correlated with smoking, alcohol consumption, elevated baseline thyroid-stimulating hormone (TSH), and increasing waist circumference, while eGFR decline associated with male sex, advanced age, prolonged diabetes duration, and elevated baseline urinary AER.

**Conclusion:** Longitudinal analysis of T2DM patients revealed an escalation in the prevalence of micro- or macroalbuminuria and renal dysfunction. Disease progression predominated over regression across the cohort. Key modifiable risk factors associated with DKD progression included tobacco use, alcohol intake, elevated baseline TSH, increasing waist circumference, male sex, age, prolonged diabetes duration, and elevated baseline urinary AER.

## 1. Introduction

Diabetic kidney disease (DKD), characterized by a progressive decline in estimated glomerular filtration rate (eGFR) and/or elevated urinary albumin excretion, is strongly associated with increased risks of end-stage renal disease and cardiovascular mortality [1, 2]. Between 1990 and 2021, diabetes-related chronic kidney disease (CKD) demonstrated alarming increases in mortality rates, age-standardized mortality rates, and disability-adjusted life years (DALYs) [3]. Current projections indicate a worsening trajectory, with estimates suggesting that, by 2050, the global burden of DKD may escalate to 95.5 million deaths and 25.7 million DALYs [3]. Clinical guidelines stratify disease severity through dual parameters: urinary albumin excretion categorized as normoalbuminuria, moderate albuminuria, or severe albuminuria, complemented by a five-stage eGFR classification system based on creatinine levels. While current recommendations emphasize annual monitoring of both eGFR and urinary albumin for early detection [4], significant knowledge gaps persist in longitudinal research. Most existing studies have insufficiently characterized eGFR trajectory patterns and dynamic albuminuria changes, with particularly limited data regarding their progression over extended follow-up periods.

DKD progression is influenced by multiple modifiable risk factors, including elevated triglyceride levels, increased resting heart rate, uncontrolled systolic blood pressure (SBP), prolonged diabetes duration, and suboptimal glycemic control [5]. Notably, existing epidemiological investigations have predominantly focused on risk factor profiling in Type 1 diabetes mellitus (T1DM) populations [5, 6]. A recent cross-sectional study of Chinese patients with T1DM revealed distinct risk factor patterns between albuminuria development and eGFR decline [6], underscoring the pathophysiological heterogeneity of renal impairment mechanisms. Long-term longitudinal studies are essential to characterize eGFR trajectory patterns and serial albuminuria changes over time. Our longitudinal cohort study was aimed at addressing these gaps by systematically evaluating 5-year trajectories of both eGFR and urinary albumin excretion rate (AER) in adults with Type 2 diabetes mellitus (T2DM). Specifically, we sought to quantify the rate of renal function decline and albuminuria progression and compare clinical, metabolic, and lifestyle profiles between “progressors” (significant eGFR and/or urinary AER stage progression) and “nonprogressors.” Our findings provide critical insights into the change between eGFR and urinary AER evolution while highlighting actionable risk factors for targeted intervention.

## 2. Methods

### 2.1. Study Design and Population

Data for this longitudinal observational cohort study came from Beijing Hospital, National Center of Gerontology, where patients had long-term illnesses and multiple diseases. Participants were consecutively recruited from March 2013 to January 2025. Inclusion criteria were as follows: (1) T2DM diagnosis and (2) admission to Beijing Hospital at least twice. The exclusion criteria for the participants were as follows: (1) absence of baseline data for UAER or serum creatinine; (2) only one measurement of urinary AER or creatinine; (3) intervals < 2.5 years between hospitalizations; (4) non-T2DM types, including T1DM, maturity-onset diabetes of the young, secondary diabetes, pancreatogenic diabetes, steroid-induced diabetes, and unclassified diabetes. Consequently, the final analysis encompassed 694 participants in total: 421 participants for the urinary AER assessment and 487 participants for the eGFR evaluation, as shown in Figure S1. This study was conducted with the approval of the Medical Ethics Committee of Beijing Hospital (Approval Number 2024BJYYEC-KY083-02).

### 2.2. Definitions

The diagnosis of T2DM was based on the American Diabetes Association (ADA)'s criteria of T2DM [7]. Hypertension was defined as either (1) SBP ≥ 140 mmHg, (2) diastolic blood pressure (DBP) ≥ 90 mmHg, or (3) a self-reported physician diagnosis of hypertension. Fatty liver disease was diagnosed via abdominal ultrasonography. Bone mineral density measurements were obtained at the lumbar spine (L1–L4) and bilateral femoral neck using dual-energy x-ray absorptiometry (DXA; GE Healthcare Lunar iDXA). Osteoporosis was diagnosed according to the World Health Organization criteria [8], defined as a *T*‐score ≤ −2.5 standard deviations below the young adult mean at either measurement site, or by self-reported history of physician-diagnosed osteoporosis. History of coronary heart disease, cerebral hemorrhage, and cerebral infarction were ascertained through self-reported physician diagnoses.

Demographic and clinical parameters including age, gender, duration of diabetes mellitus, and anthropometric measurements (weight, body mass index [BMI], waist circumference, and hip circumference) were extracted from electronic medical records. Smoking status was assessed through a standardized questionnaire item inquiring whether participants currently smoked or had completely quit, with affirmative responses to current smoking defining smokers. Regular alcohol consumption was defined as consuming alcoholic beverages more than once monthly. Blood pressure measurements were obtained following standardized protocols using a validated digital sphygmomanometer (Omron HEM-7121, Japan). After ensuring participants remained seated with back support for at least 5 min, three consecutive readings were recorded at 1-min intervals. Laboratory investigations included lipid profile, biochemical parameters, and thyroid function tests. All assays were performed using standardized laboratory methods with regularly calibrated equipment following manufacturer protocols and international quality control standards.

### 2.3. Assessment of Kidney Disease

Diagnosis of DKD was established according to the 2024 ADA Standards of Medical Care in Diabetes [4]. Urinary AER was quantified through chemiluminescent immunoassay analysis of centralized 8-h overnight urine collections. Albuminuria staging followed standardized thresholds: normoalbuminuria (AER < 20* μ*g/min), moderate albuminuria (microalbuminuria: 20–200 *μ*g/min), and severe albuminuria (macroalbuminuria: AER > 200* μ*g/min). Glomerular filtration rate (GFR) was estimated using the Chronic Kidney Disease Epidemiology Collaboration [4], with renal impairment defined as eGFR values below 90 mL/min/1.73m^2^. We conducted comparative analyses of baseline and follow-up characteristics between two progression cohorts: (1) patients transitioning from normoalbuminuria to micro- or macroalbuminuria based on UAER criteria and (2) those demonstrating renal functional decline from preserved eGFR (eGFR ≥ 90 mL/min/1.73m^2^) to impaired renal function (eGFR < 90 mL/min/1.73m^2^).

### 2.4. Statistical Analysis

Categorical variables were expressed as absolute frequencies with percentages (*n* [%]). Continuous variables with normal distributions were summarized as mean ± standard deviation, while nonnormally distributed variables were reported as median (interquartile range). To conduct intergroup comparisons, we employed the following statistical methods: For categorical variables, we used Pearson's *χ*^2^ test, and for small sample sizes, Fisher's exact test was applied. For continuous variables that were normally distributed, the independent Student *t*-test was utilized. For continuous variables that did not follow a normal distribution, the Mann–Whitney *U* test was employed. All analyses were conducted in the R statistical computing environment (Version 4.4.2, R Foundation for Statistical Computing). A two-tailed *α* level of 0.05 defined statistical significance, with *p* values < 0.05 considered statistically significant.

## 3. Results

### 3.1. Population Characteristics

Table 1 provides a comprehensive summary of the baseline and follow-up characteristics of patients. Among 694 participants with a median age of 59.00 (52.25, 67.00) years, the follow-up time was 4.53 (3.39, 6.00) years, and 258 (37.2%) participants were female. Compared to baseline measurements, follow-up patients exhibited older age, reduced body weight, and higher prevalence of hypertension, fatty liver, coronary heart disease, osteoporosis, cerebral hemorrhage history, cerebral infarction history, diabetic peripheral neuropathy, and diabetic retinopathy. Follow-up assessments revealed elevated heart rate, SBP, HDL-c, uric acid, and creatinine levels, alongside decreased LDL-c, total cholesterol, triglycerides, AST, albumin, and eGFR.

### 3.2. Classification and Group Changes of Urinary AER

**Figure 1:**
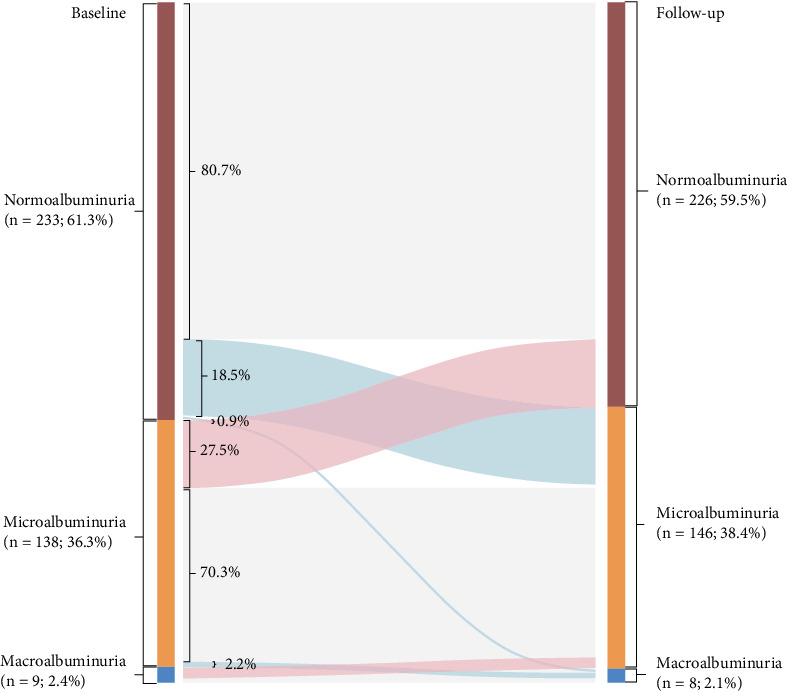
Longitudinal reclassification patterns of urinary AER severity (*n* = 421) were modeled using a Sankey plot. AER, albumin excretion rate.

To evaluate the natural progression of DKD, our analysis focused exclusively on patients without confounding comorbidities and with confirmatory albuminuria testing at both baseline and follow-up.  Figure 1 presents the Sankey diagram detailing longitudinal transitions in albuminuria categories after a median follow-up time of 4.5 (3.4, 6.0) years. In the final analytical cohort of 421 participants, baseline-to-follow-up albuminuria distribution demonstrated dynamic shifts: macroalbuminuria prevalence decreased marginally from 2.4% to 2.1%, microalbuminuria increased from 36.3% to 38.4%, and normoalbuminuria declined from 61.3% to 59.5%. Transition patterns revealed distinct trajectories: Among 299 baseline normoalbuminuric participants (61.3%), 18.5% (*n* = 55) progressed to microalbuminuria, while 0.9% (*n* = 3) transitioned directly to macroalbuminuria. Of 177 patients with baseline microalbuminuria (36.3%), 27.5% (*n* = 49) regressed to normoalbuminuria, whereas 2.2% (*n* = 4) progressed to macroalbuminuria. Notably, 66.7% (*n* = 6) of the nine baseline macroalbuminuric patients regressed to microalbuminuria, with no cases reverting to normoalbuminuria. Cumulatively, 9.9% (*n* = 48) of patients exhibited disease progression to higher albuminuria stages. Net changes demonstrated a 1.8% absolute reduction in normoalbuminuria prevalence alongside a 1.8% aggregate increase in micro-/macroalbuminuria proportions. A strong graded relationship emerged between baseline albuminuria severity and macroalbuminuria progression risk (*p* < 0.001).

Table 2 presents baseline characteristics of the 421 participants eligible for urinary AER analysis. Compared to those maintaining normal AER, progressors were more likely to smoke, drink, had a higher baseline thyroid-stimulating hormone (TSH), a lower prevalence of diabetic peripheral neuropathy, and a smaller baseline waist circumference but a relatively high waist circumference at follow-up, indicating an increase in waist size. At follow-up, progressors were more likely to smoke and had a lower level of HbA1c.

### 3.3. Classification and Group Changes of eGFR

**Figure 2:**
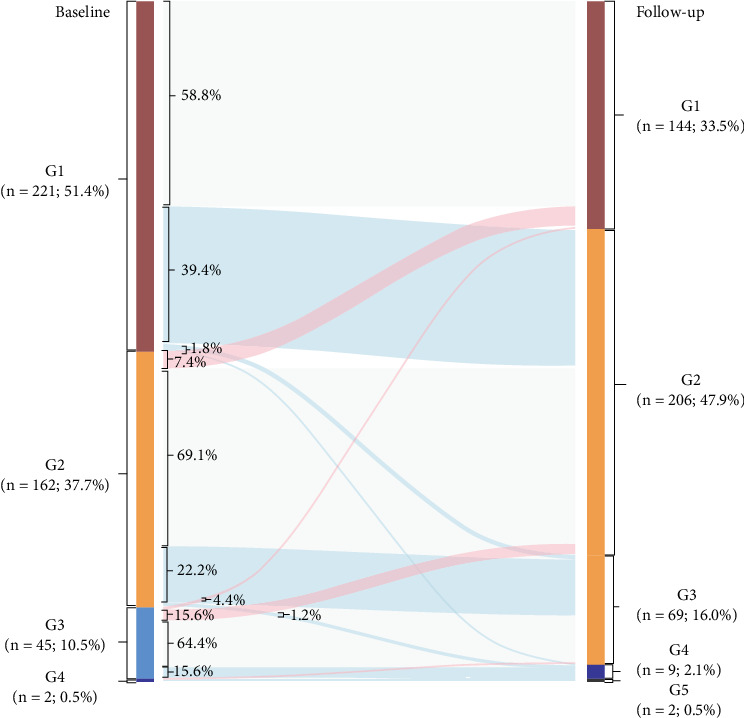
Longitudinal reclassification patterns of eGFR severity (*n* = 487) were modeled using a Sankey plot. eGFR, estimated glomerular filtration rate.

The patient flow is depicted in the Sankey diagram (Figure 2) after a median follow-up time of 4.5 years. Among the 487 patients in the subgroup, the distribution of groups at baseline and follow-up was as follows: Stage G1 accounted for 51.4% and 33.5%, Stage G2 for 37.7% and 47.9%, Stage G3 for 10.5% and 16.0%, Stage G4 for 0.5% and 2.1%, and Stage G5 for 0% and 0.5%, respectively. In the Stage G1 group, 221 participants (51.4%) were present at baseline, with 39.4% progressing to Stage G2, 1.8% progressing to Stage G3, and 1.8% progressing to Stage G4 at follow-up. In the Stage G2 group, 162 participants (37.7%) were present at baseline, with 7.4% regressing to Stage G1, 22.2% progressing to Stage G3, and 1.2% progressing to Stage G4 at follow-up. In the Stage G3 group, 45 participants (10.5%) were present at baseline, with 4.4% regressing to Stage G1, 15.6% regressing to Stage G2, 13.3% progressing to Stage G4, and 2.2% progressing to Stage G5 at follow-up. In the Stage G4 group, two participants (0.5%) were present at baseline, with 50.0% regressing to Stage G3 and 50.0% progressing to Stage G5 at follow-up. Overall, compared with baseline, 137 patients (32.5%) developed worsened renal function at follow-up. The proportion of patients with impaired eGFR increased, while the proportion of patients with normal eGFR decreased. The risk of developing Stage G5 at follow-up was significantly related to lower eGFR at baseline (*p* < 0.001).

Table 3 presents the baseline demographic and clinical characteristics of the 487 participants included in the eGFR analysis. Compared with those who maintained normal eGFR levels, individuals in the progression group were more likely to be male and older, with longer diabetes duration and higher baseline urinary AER. At follow-up, the progression group was also more likely to smoke and drink alcohol, had a longer duration of follow-up, and exhibited lower levels of serum albumin and aspartate aminotransferase. After adjusting for sex, significant differences persisted in age, diabetes duration, follow-up time, and albumin levels.

### 3.4. Subgroup Analysis

We performed subgroup analyses to explore progression characteristics and risk factor differences by gender and disease stage. Table S1 shows baseline characteristics of females in the eGFR analysis. Compared to the normal eGFR group, the progression group had older age, longer diabetes duration, and higher hypertension prevalence at baseline. Table S2 shows baseline characteristics of males. The progression group had older age and longer diabetes duration versus controls. Table S3 presents characteristics of participants with diabetes duration < 10 years. The progression group showed older age, male predominance, longer diabetes duration, and lower heart rate at baseline. Table S4 shows characteristics of those with 10–20 years' diabetes duration. The progression group had a higher male proportion and fatty liver prevalence at baseline.

## 4. Discussion

This study revealed an overall worsening of DKD severity over time. Among patients with T2DM and a median diabetes duration of 12 years, the prevalence of normoalbuminuria, microalbuminuria, and macroalbuminuria was 61.3%, 36.6%, and 2.4%, respectively. After a follow-up period of 4.5 years, 9.9% (*n* = 48) of patients exhibited disease progression to higher stages of albuminuria. A strong graded relationship emerged between baseline albuminuria severity and the risk of macroalbuminuria progression. The prevalence of G1, G2, G3, and G4 was 51.4%, 37.7%, 10.5%, and 0.5%, respectively; 32.5% (*n* = 137) developed worsened renal function. The risk of developing Stage G5 at follow-up was significantly associated with lower eGFR at baseline.

Some studies have examined the progression of albuminuria stages and eGFR trajectories over time. Our findings align with seminal data from the UK Prospective Diabetes Study (UKPDS), where extended 15-year surveillance revealed cumulative renal complications in treatment-naïve patients: 38% developed microalbuminuria, while 29% exhibited reduced eGFR (eGFR ≤ 60 mL/min/1.73m^2^) [9]. Another finding revealed progressive microalbuminuria escalation in newly diagnosed T2DM: 7.3% baseline prevalence increased to 17.3% (5-year), 24.9% (10-year), and 28.0% (15-year) [10]. Consistent with our findings, a cross-sectional study identified older age, male sex, smoking, alcohol use, excess weight, and the cardiometabolic cluster of diabetes, hypertension, dyslipidemia, and hyperuricemia as independent determinants of both reduced eGFR and albuminuria [11]. A separate retrospective cohort observed a 14.4% incidence of rapid eGFR decline over a median follow-up of 29 months; predictors included longer diabetes duration, elevated SBP, higher serum uric acid, albuminuria, and lower baseline eGFR [12].

Tobacco use and alcohol consumption were confirmed as established risk factors for elevated AER progression [13, 14]. Notably, our analysis revealed that progressors demonstrated lower baseline waist circumference yet a relatively high waist circumference at follow-up. No significant between-group differences in BMI or body weight were observed at baseline or during follow-up. This dynamic waist circumference escalation suggested that central adiposity redistribution may potentiate renal endothelial dysfunction. Emerging evidence implicates central adiposity as an independent renal risk predictor: Dutch cohort data revealed elevated microalbuminuria risks not only in obese/overweight individuals with android fat distribution but also in lean subjects exhibiting central adiposity [15]. This risk continuum persists across BMI strata, as multinational studies demonstrate waist circumference-associated microalbuminuria gradients at equivalent BMI levels [16]. Bioelectrical impedance-derived visceral fat area measurements further corroborate this association, showing significant correlations with urinary albumin–creatinine ratio (UACR) elevation [17]. Mechanistically, ectopic fat deposition triggers endothelial dysfunction via proinflammatory cytokine cascades and prothrombotic states, establishing visceral obesity as a key mediator of albuminuria pathogenesis [16].

The interplay between thyroid function parameters and DKD progression remains mechanistically contested. Our data identified that progressors of urinary AER were more likely to have an elevated baseline TSH. A study on T2DM revealed a positive correlation between TSH and UACR. Specifically, in individuals with free triiodothyronine (FT3) levels ≤ 4.30 pmol/L for males and ≤ 3.99 pmol/L for females, the risk of renal disease progression in patients with T2DM was significantly increased [18]. Thyroid hormones play a role in glucose metabolism, and the prevalence of thyroid dysfunction is higher among diabetic patients compared to healthy individuals [19]. Thyroid hormones can influence vasodilation, modulate endothelial function, and regulate homeostasis signaling pathways [20], thereby reducing endothelial dysfunction and decreasing urinary microalbuminuria. Research has demonstrated that subclinical hypothyroidism emerged as an independent DKD predictor [21], suggesting that thyroid hormone optimization may attenuate albuminuria progression via endothelial stabilization.

In our research, compared with those who maintained normal eGFR levels, individuals in the progression group were more likely to be male and older, with longer diabetes duration and higher baseline urinary AER. Previous studies have shown that the high risk of CKD progression is positively correlated with male gender and the duration of diabetes [6]. Compared to males, females have a lower risk of CKD progression. Possible explanations for the difference in CKD progression risk between males and females include the protective effects of endogenous estrogen and the different impacts of gender on lifestyle and traditional risk factors [22]. In the DCCT/EDIC cohort, patients with microalbuminuria had an average annual eGFR decline of 1.2% and a 15% 10-year cumulative incidence of eGFR decline, while those with macroalbuminuria experienced an average annual eGFR loss of 5.7% and a 32% 10-year cumulative incidence of eGFR decline [23]. This suggests that reducing urinary albumin can slow the progression of DKD and have a protective effect on the kidneys.

In the urinary AER analysis, both progressors and nonprogressors exhibited comparable baseline HbA1c levels, though values were elevated in both groups. The emergence of albuminuria or progression of CKD may have influenced clinical decision-making, prompting clinicians to adopt distinct antihyperglycemic regimens that potentially contributed to observed disparities in follow-up glycemic control. At follow-up, the eGFR progression group showed lower serum albumin levels. When renal function declines, especially with glomerular filtration membrane damage as in diabetic nephropathy, increased glomerular permeability leads to urinary albumin loss, causing hypoalbuminemia. Moreover, reduced renal function is linked to heightened systemic inflammation and oxidative stress. Inflammatory mediators can suppress hepatic albumin synthesis and accelerate albumin catabolism, thereby lowering serum albumin levels.

Current ADA guideline recommends annual assessment of urinary albumin and eGFR for all individuals with Type 2 diabetes [4]. In those diagnosed with CKD, monitoring frequency should be escalated to 1–4 times annually based on disease stage. Given the risks associated with DKD, our findings suggest intensified urinary albumin and eGFR evaluation for T2DM patients exhibiting any of the following characteristics: male sex, elevated waist circumference, smoking, alcohol consumption, or subclinical hypothyroidism. Angiotensin-converting enzyme inhibitors (ACEIs) or angiotensin receptor blockers (ARBs) remain first-line antihypertensive agents for diabetic patients with hypertension. Additionally, regular monitoring of serum potassium and creatinine is advised for individuals prescribed ACEIs, ARBs, or mineralocorticoid receptor antagonists (MRAs). Regarding glucose-lowering therapies, SGLT2 inhibitors and GLP-1 receptor agonists demonstrate clinically validated renoprotective benefits [4].

Our study has several limitations. First, we assessed urinary albumin levels via urinary AER, rather than UACR, which was measured in untimed “spot” specimens. Existing literature demonstrates moderate correlation between the two methods (*r* = 0.62) [24]. A note that Kidney Disease Improving Global Outcomes (KDIGO) guidelines acknowledge both timed collections and UACR as valid diagnostic methods for albuminuria staging [25, 26], though UACR is preferred for routine use. Importantly, the consistent application of the 8-h AER method across all included patients ensures the internal validity of the associations observed within our study cohort. Second, the inclusion of a single-center hospitalized T2DM cohort introduces potential selection bias, limiting generalizability to other demographic groups. The selection bias inherent in studying hospitalized patients limits generalizability to nonhospitalized or community-based T2DM cohorts. There is a need for future validation in larger, multicenter prospective studies encompassing broader patient demographics (including community settings) to confirm the generalizability of our findings. Nevertheless, this cohort provides valuable insights into associations within a well-characterized, high-risk inpatient T2DM population managed under standardized protocols at a major referral center. Future prospective multicenter studies incorporating community and outpatient cohorts are warranted to verify these findings. Third, the lack of medication data precludes assessment of whether ACEIs/ARBs or MRAs modified DKD progression trajectories compared to untreated patients. Patients with worse albuminuria are more likely to be prescribed renin-angiotensin-aldosterone system inhibitor (RAASi). The observed associations between biomarkers and DKD progression may be influenced by unmeasured RAASi use and cannot establish causality independent of this treatment. Future prospective studies must systematically collect granular data on RAASi (and SGLT2 inhibitors) usage, including dose, duration, and adherence, to accurately evaluate novel biomarkers or risk factors within the context of contemporary guideline-directed medical therapy.

## 5. Conclusion

Our results demonstrate progressive DKD severity in T2DM, with disease progression correlating with smoking, alcohol consumption, elevated baseline TSH, increasing waist circumference, male sex, age, prolonged diabetes duration, and heightened baseline urinary AER.

## Figures and Tables

**Table 1:** Baseline and follow-up characteristics of Type 2 diabetes patients. Data are presented as mean ± SD, number (percentage), or median (interquartile range). AER, albumin excretion rate; eGFR, estimated glomerular filtration rate; BMI, body mass index; SBP, systolic blood pressure; HDL-c, high-density lipoprotein cholesterol; LDL-c, low-density lipoprotein cholesterol; FPG, fasting glucose; AST, aspartate aminotransferase; TSH, thyroid-stimulating hormone; FT3, free triiodothyronine; FT4, free thyroxine.

**Variable**	**Baseline**	**Follow-up**
Female	258 (37.2%)	258 (37.2%)
Age (years)	59.00 (52.25, 67.00)	65.00 (57.00, 71.00)^a^
Diabetes duration (years)	12 (6, 18)	17 (10, 22)^a^
Follow-up time (years)	—	4.53 (3.39, 6.00)
Weight (kg)	71.00 (63.00, 80.00)	70.10 (62.12, 79.97)^a^
BMI (kg/m^2^)	25.15 (23.15, 27.68)	25.06 (23.13, 27.39)
Waist circumference (cm)	94.80 ± 10.44	95.60 ± 11.12
Hip circumference (cm)	100.20 ± 8.32	100.84 ± 8.76
Waist-to-hip ratio	0.95 ± 0.06	0.95 ± 0.07
Smoking	313 (45.1%)	315 (45.4%)
Drinking	300 (43.2%)	296 (42.7%)
Hypertension	396 (57.1%)	446 (64.3%)^a^
Fatty liver	157 (22.6%)	252 (36.3%)^a^
Coronary heart disease	171 (24.6%)	227 (32.7%)^a^
Osteoporosis	57 (8.2%)	102 (14.7%)^a^
History of cerebral hemorrhage	6 (0.9%)	18 (2.6%)^a^
History of cerebral infarction	104 (15%)	152 (21.9%)^a^
Diabetic peripheral neuropathy	462 (66.6%)	519 (74.8%)^a^
Diabetic retinopathy	198 (28.5%)	236 (34%)^a^
Heart rate	72 (70,78)	74 (71, 78)^a^
SBP (mmHg)	128 (120, 139)	130 (121, 140)^a^
HDL-c (mmol/L)	1.03 (0.88, 1.22)	1.07 (0.90, 1.29)^a^
LDL-c (mmol/L)	2.40 (1.87, 3.05)	2.20 (1.69, 2.76)^a^
Total cholesterol (mmol/L)	4.12 (3.49, 4.79)	3.90 (3.29, 4.58)^a^
Triglyceride (mmol/L)	1.31 (0.89, 2.12)	1.26 (0.88, 1.95)^a^
FPG (mmol/L)	7.60 (6.00, 9.65)	7.20 (5.80, 9.10)
HbA1c (%)	8.70 (7.50, 10.00)	8.60 (7.40, 9.70)
Uric acid (*μ*mol/L)	325 (272, 388)	342 (279, 415)^a^
AST (U/L)	18 (15, 23)	17 (14, 22)^a^
Albumin (g/L)	40.74 ± 3.03	39.34 ± 3.35^a^
Urinary AER (*μ*g/min)	14.20 (7.60, 41.50)	13.95 (7.70, 35.35)
eGFR (mL/min/1.73m^2^)	92.29 (73.99, 100.91)	83.72 (65.78, 95.31)^a^
Creatinine (mg/dL)	62 (52, 75)	67 (57, 81)^a^
TSH (*μ*IU/mL)	1.88 (1.23, 2.88)	1.87 (1.18, 2.76)
FT3 (pg/mL)	2.93 (2.72, 3.18)	2.95 (2.66, 3.15)
FT4 (ng/dL)	1.17 (1.06, 1.31)	1.17 (1.06, 1.28)

^a^ *p* < 0.05 versus baseline.

**Table 2:** Baseline and follow-up characteristics of Type 2 diabetes patients: No progression versus progression to micro- or macroalbuminuria. Data are presented as mean ± SD, number (percentage), or median (interquartile range). BMI, body mass index; SBP, systolic blood pressure; HDL-c, high-density lipoprotein cholesterol; LDL-c, low-density lipoprotein cholesterol; FPG, fasting glucose; AST, aspartate aminotransferase; TSH, thyroid-stimulating hormone; FT3, free triiodothyronine; FT4, free thyroxine.

**Variable**	**No progression (** **n** = 188**)**		**Progression (** **n** = 45**)**	
	**Baseline**	**Follow-up**	**Baseline**	**Follow-up**
Female	—	69 (36.7%)	—	11 (24.4%)
Age (years)	59.89 ± 11.39	64.83 ± 11.41	56.58 ± 10.53	61.87 ± 10.79
Diabetes duration (years)	12.53 ± 8.12	16.61 ± 8.55	11.02 ± 6.17	17.36 ± 6.80
Follow-up time (years)	—	4.87 (3.64, 6.31)	—	5.64 (3.87, 6.58)
Weight (kg)	72.00 (63.25, 80.50)	70.00 (61.75, 80.00)	70.25 (61.50, 79.03)	70.00 (63.00, 80.25)
BMI (kg/m^2^)	25.26 (23.57, 27.50)	25.01 (23.24, 27.43)	25.34 (21.80, 27.03)	25.23 (21.97, 27.43)
Waist circumference (cm)	96.08 ± 8.88	96.47 ± 9.89	89.91 ± 9.49^a^	95.67 ± 10.29
Hip circumference (cm)	101.68 ± 6.69	101.42 ± 8.28	97.91 ± 10.39	101.53 ± 8.49
Waist-to-hip ratio	0.94 ± 0.05	0.95 ± 0.06	0.91 ± 0.04	0.95 ± 0.06
Smoking	79 (42.0%)	77 (41.0%)	27 (60.0%)^a^	27 (60.0%)^a^
Drinking	77 (41.0%)	81 (43.1%)	27 (60.0%)^a^	26 (57.8%)
Hypertension	117 (62.2%)	125 (66.8%)	29 (64.4%)	31 (68.9%)
Fatty liver	44 (23.4%)	69 (36.7%)	13 (28.9%)	17 (37.8%)
Coronary heart disease	45 (23.9%)	66 (35.1%)	10 (22.2%)	14 (31.1%)
Osteoporosis	12 (6.4%)	28 (14.9%)	3 (6.7%)	4 (8.9%)
History of cerebral hemorrhage	6 (3.2%)	8 (4.3%)	0 (0.0%)	1 (2.2%)
History of cerebral infarction	33 (17.6%)	51 (27.1%)	6 (13.3%)	11 (24.4%)
Diabetic peripheral neuropathy	124 (66.0%)	139 (73.9%)	21 (46.7%)^a^	30 (66.7%)
Diabetic retinopathy	50 (26.6%)	60 (31.9%)	10 (22.2%)	14 (31.1%)
Heart rate	72 (70, 76)	74 (71, 78)	72 (70, 78)	74 (72, 78)
SBP (mmHg)	129.84 ± 15.40	132.26 ± 17.09	134.04 ± 19.26	129.22 ± 17.17
HDL-c (mmol/L)	1.01 ± 0.21	1.06 ± 0.25	1.12 ± 0.27	1.11 ± 0.26
LDL-c (mmol/L)	2.35 ± 0.85	2.28 ± 0.86	2.73 ± 0.88	2.24 ± 0.9
Total cholesterol (mmol/L)	3.99 ± 0.95	3.97 ± 1.12	4.38 ± 1.00	3.92 ± 1.13
Triglyceride (mmol/L)	1.42 (1.02, 2.26)	1.46 (0.95, 2.20)	1.28 (0.75, 2.03)	1.33 (0.94, 1.79)
FPG (mmol/L)	7.80 (6.65, 9.40)	7.25 (5.93, 8.70)	7.90 (5.90, 10.90)	6.40 (5.65, 8.48)
HbA1c (%)	9.08 ± 2.21	8.91 ± 1.79	8.78 ± 1.82	8.07 ± 1.38^a^
Uric acid (*μ*mol/L)	366.46 ± 225.88	413.24 ± 374.37	352.06 ± 120.40	460.59 ± 363.82
AST (U/L)	19.00 (15.50, 24.00)	18.00 (15.00, 22.00)	17.00 (16.00, 21.00)	18.00 (14.00, 23.00)
Albumin (g/L)	40.33 ± 3.62	39.37 ± 3.03	42.06 ± 2.86	40.11 ± 3.07
TSH (*μ*IU/mL)	1.67 (1.17, 2.76)	1.73 (1.06, 2.54)	1.88 (1.45, 3.90)^a^	2.13 (1.07, 2.75)
FT3 (pg/mL)	1.24 ± 0.29	2.94 ± 0.45	1.20 ± 0.23	2.84 ± 0.34
FT4 (ng/dL)	3.02 ± 1.05	1.22 ± 0.19	2.95 ± 0.39	1.19 ± 0.22

^a^ *p* < 0.05 versus no progression at the same time point.

**Table 3:** Baseline and follow-up characteristics of Type 2 diabetes patients: No progression versus progression to impaired eGFR. Data are presented as mean ± SD, number (percentage), or median (interquartile range). eGFR, estimated glomerular filtration rate; BMI, body mass index; SBP, systolic blood pressure; HDL-c, high-density lipoprotein cholesterol; LDL-c, low-density lipoprotein cholesterol; FPG, fasting glucose; AST, aspartate aminotransferase; AER, albumin excretion rate; TSH, thyroid-stimulating hormone; FT3, free triiodothyronine; FT4, free thyroxine.

**Variable**	**No progression (** **n** = 130**)**		**Progression (** **n** = 91**)**	
	**Baseline**	**Follow-up**	**Baseline**	**Follow-up**
Female	—	74 (56.9%)	—	30 (33.0%)^a^
Age (years)	53.50 ± 11.76	58.08 ± 11.90	60.60 ± 8.37^a,b^	65.60 ± 8.37^a,b^
Diabetes duration (years)	9 (4, 15)	13 (7, 20)	12 (7, 18)^a,b^	18 (10, 24)^a,b^
Follow-up time (years)	—	4.40 (3.35, 5.88)	—	4.93 (3.85, 6.12)^b^
Weight (kg)	70.00 (60.00, 79.10)	70.00 (61.25, 79.75)	70.00 (63.25, 77.75)	70.00 (60.50, 78.50)
BMI (kg/m^2^)	25.18 (23.05, 27.40)	25.42 (22.52, 27.39)	24.91 (22.89, 27.36)	24.86 (22.84, 27.16)
Waist circumference (cm)	92.90 ± 10.30	95.21 ± 10.03	94.84 ± 9.10	95.02 ± 8.21
Hip circumference (cm)	98.45 ± 6.86	99.81 ± 7.22	99.26 ± 7.66	99.66 ± 5.37
Waist-to-hip ratio	0.94 ± 0.06	0.96 ± 0.07	0.96 ± 0.06	0.95 ± 0.06
Smoking	45 (34.6%)	42 (32.3%)	42 (46.2%)	48 (52.7%)^a^
Drinking	47 (36.2%)	42 (32.3%)	40 (44.0%)	43 (47.3%)^a^
Hypertension	66 (50.8%)	72 (55.4%)	52 (57.1%)	58 (63.7%)
Fatty liver	45 (34.6%)	56 (43.1%)	26 (28.6%)	44 (48.4%)
Coronary heart disease	25 (19.2%)	34 (26.2%)	17 (18.7%)	32 (35.2%)
Osteoporosis	16 (12.3%)	22 (16.9%)	6 (6.6%)	12 (13.2%)
History of cerebral hemorrhage	1 (0.8%)	1 (0.8%)	0 (0.0%)	2 (2.2%)
History of cerebral infarction	15 (11.5%)	20 (15.4%)	15 (16.5%)	21 (23.1%)
Diabetic peripheral neuropathy	84 (64.6%)	87 (66.9%)	61 (67.0%)	67 (73.6%)
Diabetic retinopathy	40 (30.8%)	39 (30.0%)	22 (24.2%)	31 (34.1%)
Heart rate	74 (72, 78)	74 (72, 78)	74 (71, 78)	76 (71, 78)
SBP (mmHg)	130.55 ± 15.37	132.05 ± 17.24	130.76 ± 16.95	133.03 ± 15.85
HDL-c (mmol/L)	1.03 (0.88, 1.20)	1.06 (0.93, 1.29)	1.06 (0.88, 1.22)	1.09 (0.93, 1.30)
LDL-c (mmol/L)	2.45 (2.08, 3.07)	2.26 (1.81, 2.78)	2.31 (1.75, 3.06)	2.22 (1.74, 2.55)
Total cholesterol (mmol/L)	4.32 ± 1.06	4.08 ± 1.09	4.13 ± 0.94	3.93 ± 0.98
Triglyceride (mmol/L)	1.48 (1.02, 2.26)	1.37 (0.97, 2.16)	1.42 (0.93, 2.39)	1.31 (0.93, 2.06)
FPG (mmol/L)	7.70 (6.00, 9.93)	7.00 (5.62, 9.20)	7.50 (6.30, 10.10)	6.80 (5.70, 8.85)
HbA1c (%)	9.00 (7.47, 10.10)	8.50 (7.20, 9.80)	8.80 (7.20, 10.70)	8.30 (7.12, 9.67)
Uric acid (*μ*mol/L)	318.60 ± 85.08	386.17 ± 429.30	321.68 ± 76.63	362.99 ± 150.55
AST (U/L)	18.50 (16.00, 24.75)	18.00 (15.00, 23.00)	17.00 (14.00, 21.50)	16.00 (14.00, 20.00)^a^
Albumin (g/L)	41.54 ± 2.55	40.36 ± 3.31	40.88 ± 2.59	39.30 ± 3.30^a,b^
Urinary AER (*μ*g/min)	32.24 ± 35.98	38.98 ± 47.12	60.32 ± 128.03^b^	26.90 ± 34.00
TSH (*μ*IU/mL)	1.85 (1.37, 3.00)	1.85 (1.21, 2.86)	1.71 (1.09, 2.68)	2.08 (1.27, 3.12)
FT3 (pg/mL)	2.99 ± 0.41	3.10 ± 1.17	3.04 ± 0.36	2.93 ± 0.37
FT4 (ng/dL)	1.20 ± 0.18	1.20 ± 0.19	1.20 ± 0.20	1.20 ± 0.20

^a^ *p* < 0.05 versus no progression at the same time point.

^b^ *p* < 0.05 after adjustment for sex.

## Data Availability

The data that support the findings of this study are available on request from the corresponding authors. The data are not publicly available due to privacy or ethical restrictions.

## Supporting Information

Supporting Information Additional supporting information can be found online in the Supporting Information section. Figure S1. Flowchart of participant selection. Table S1. Baseline and follow-up characteristics of Type 2 diabetes female patients. Table S2. Baseline and follow-up characteristics of Type 2 diabetes male patients. Table S3. Baseline and follow-up characteristics of Type 2 diabetes patients and diabetes duration < 10 years. Table S4. Baseline and follow-up characteristics of Type 2 diabetes patients and a diabetes duration of 10–20 years.
