# Supplementary material for: Longitudinal Trajectories of Albuminuria and eGFR in Type 2 Diabetes Mellitus: Natural Progression of Diabetic Kidney Disease
Source: J Diabetes Res. 2025 Aug 18;2025:9269085. doi: 10.1155/jdr/9269085 (PMC12377932; doi:10.1155/jdr/9269085)
Supplement: Supporting Information — Additional supporting information can be found online in the Supporting Information section. Figure S1. Flowchart of participant selection. Table S1. Baseline and follow-up characteristics of Type 2 diabetes female patients. Table S2. Baseline and follow-up characteristics of Type 2 diabetes male patients. Table S3. Baseline and follow-up characteristics of Type 2 diabetes patients and diabetes duration < 10 years. Table S4. Baseline and follow-up characteristics of Type 2 diabetes patients and a diabetes duration of 10–20 years. [file 9269085.f1.docx]

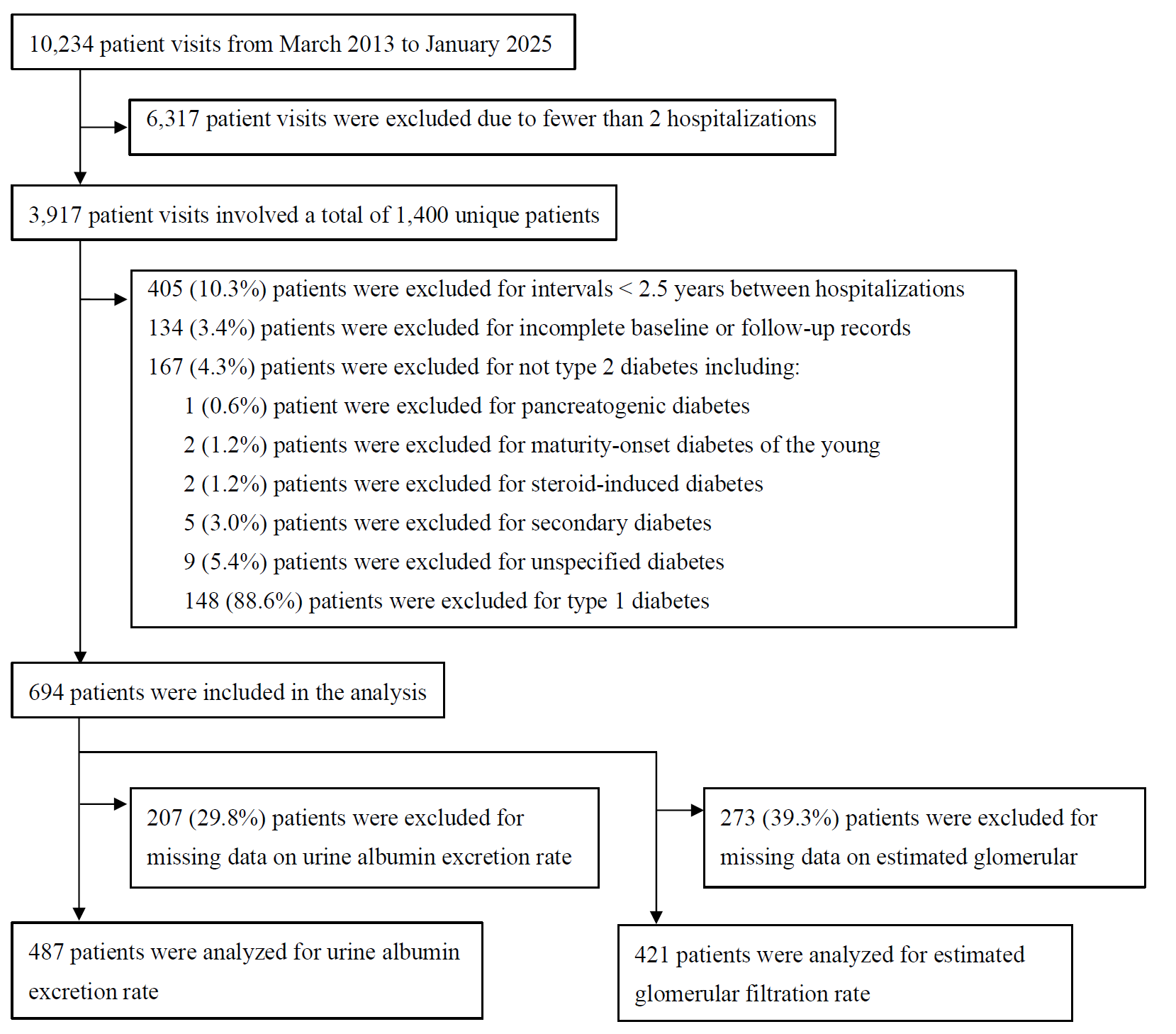
**Supplementary Figure 1**. Flowchart of Participant Selection.

**Supplementary Table 1**. Baseline and Follow-Up Characteristics of Type 2 Diabetes Female Patients: No Progression vs. Progression to Impaired eGFR. Data are presented as mean ± SD, number (percentage), or median (interquartile range). eGFR, estimated glomerular filtration rate; BMI, body mass index; SBP, systolic blood pressure; HDL-c, high-density lipoprotein cholesterol; LDL-c, low density lipoprotein cholesterol; FPG, fasting glucose; AST, aspartate aminotransferase; AER, albumin excretion rate; TSH, thyroid-stimulating hormone; FT3, free triiodothyronine; FT4, free thyroxine. ^a^p < 0.05 vs no progression at same time point.

| Variable | No progression (n = 74) | | Progression (n = 30) | |
| --- | --- | --- | --- | --- |
|  | Baseline | Follow-up | Baseline | Follow-up |
| Age (y) | 58.34±9.99 | 63.12±10.12 | 64.90±6.76^a^ | 70.13±6.85^a^ |
| Diabetes duration (y) | 10.00(5.00,17.00) | 15.00(9.00,21.00) | 16.00(8.00,18.00)^a^ | 21.00(14.25,25.00)^a^ |
| Follow-up time (y) | ― | 4.71(3.68,5.97) | ― | 5.37(4.18,6.22) |
| Weight (kg) | 68.00(57.50,77.25) | 65.75(54.75,74.00) | 67.75(59.25,74.75) | 63.50(58.00,70.00) |
| BMI (kg/m^2^) | 25.03(22.11,27.38) | 25.01(21.86,27.05) | 25.24(23.09,27.78) | 24.03(22.36,27.07) |
| Waist  circumference (cm) | 90.96±9.98 | 96.50±7.50 | 93.67±9.31 | 90.20±3.33^a^ |
| Hip  circumference (cm) | 97.55±7.05 | 100.09±7.19 | 97.68±6.88 | 98.30±5.50 |
| Waist-to-hip ratio | 0.94±0.06 | 0.97±0.06 | 0.96±0.07 | 0.92±0.04^a^ |
| Smoking | 6(8.1%) | 5(6.8%) | 2(6.7%) | 4(13.3%) |
| Drinking | 4(5.4%) | 4(5.4%) | 1(3.3%) | 2(6.7%) |
| Hypertension | 41(55.4%) | 47(63.5%) | 24(80%)^a^ | 24(80%) |
| Fatty liver | 24(32.4%) | 32(43.2%) | 9(30%) | 13(43.3%) |
| Coronary heart  disease | 16(21.6%) | 23(31.1%) | 5(16.7%) | 12(40%) |
| Osteoporosis | 13(17.6%) | 18(24.3%) | 4(13.3%) | 8(26.7%) |
| History of cerebral  hemorrhage | 1(1.4%) | 1(1.4%) | 0(0.0%) | 0(0.0%) |
| History of cerebral  infarction | 10(13.5%) | 11(14.9%) | 4(13.3%) | 8(26.7%) |
| Diabetic peripheral neuropathy | 45(60.8%) | 49(66.2%) | 24(80%) | 22(73.3%) |
| Diabetic retinopathy | 24(32.4%) | 27(36.5%) | 6(20%) | 12(40%) |
| Heart rate | 74.00(72.00,78.00) | 75.00(72.00,78.00) | 74.00(71.25,76.00) | 75.00(70.00,78.00) |
| SBP (mmHg) | 133.51±15.89 | 132.82±18.01 | 131.33±14.77 | 131.80±15.41 |
| HDL-c (mmol/L) | 1.07(0.97,1.33) | 1.15(0.99,1.37) | 1.10(1.00,1.27) | 1.23(1.02,1.36) |
| LDL-c (mmol/L) | 2.43(1.84,2.95) | 2.26(1.83,2.76) | 2.72(2.06,3.24) | 2.36(1.93,2.69) |
| Total cholesterol  (mmol/L) | 4.35±1.16 | 4.18±1.18 | 4.45±0.98 | 4.25±0.99 |
| Triglyceride (mmol/L) | 1.35(0.91,2.19) | 1.28(0.90,2.07) | 1.66(1.10,2.32) | 1.62(1.05,2.14) |
| FPG (mmol/L) | 7.50(5.73,9.47) | 7.20(5.95,9.40) | 7.10(6.17,9.15) | 7.65(6.40,10.28) |
| HbA1c (%) | 8.80(7.40,9.90) | 8.80(7.23,9.97) | 8.50(7.60,10.60) | 7.95(7.47,9.68) |
| Uric acid (μmol/L) | 297.85±75.99 | 376.72±504.40 | 318.53±61.61 | 391.33±225.94 |
| AST (U/L) | 18.00(16.00,24.00) | 18.00(15.00,23.00) | 19.00(16.00,23.50) | 18.50(15.00,21.75) |
| Albumin (g/L) | 41.42±2.44 | 39.84±3.56 | 41.03±2.67 | 39.17±3.52 |
| Urinary AER  (μg/min) | 39.95±41.70 | 41.06±42.31 | 66.44±85.84 | 30.34±29.66 |
| TSH (μIU/mL) | 1.87(1.46,3.12) | 1.85(1.04,2.40) | 1.52(1.03,2.13) | 1.85(1.15,2.80) |
| FT3 (pg/mL) | 3.03±0.39 | 3.20±1.55 | 3.11±0.39 | 2.74±0.47 |
| FT4 (ng/dL) | 1.19±0.19 | 1.21±0.21 | 1.23± 0.20 | 1.18±0.21 |

**Supplementary Table 2**. Baseline and Follow-Up Characteristics of Type 2 Diabetes Male Patients: No Progression vs. Progression to Impaired eGFR. Data are presented as mean ± SD, number (percentage), or median (interquartile range). eGFR, estimated glomerular filtration rate; BMI, body mass index; SBP, systolic blood pressure; HDL-c, high-density lipoprotein cholesterol; LDL-c, low density lipoprotein cholesterol; FPG, fasting glucose; AST, aspartate aminotransferase; AER, albumin excretion rate; TSH, thyroid-stimulating hormone; FT3, free triiodothyronine; FT4, free thyroxine. ^a^p < 0.05 vs no progression at same time point.

| Variable | No progression (n = 130) | | Progression (n = 91) | |
| --- | --- | --- | --- | --- |
|  | Baseline | Follow-up | Baseline | Follow-up |
| Age (y) | 47.11±10.89 | 51.43±10.81 | 58.49±8.31^a^ | 63.38±8.20^a^ |
| Diabetes duration (y) | 7.50(3.75,12.00) | 10.00(7.00,17.25) | 10.50(6.00,18.00)^a^ | 16.00(10.00,23.00)^a^ |
| Follow-up time (y) | ― | 3.92(3.01,5.69) | ― | 4.46(3.66,6.00) |
| Weight (kg) | 75.00(65.00,80.00) | 76.50(66.75,81.75) | 70.00(64.50,79.00) | 73.00(63.00,80.00) |
| BMI (kg/m^2^) | 25.61(23.76,27.68) | 26.40(24.18,27.95) | 24.65(22.86,27.04) | 25.09(23.24,26.99) |
| Waist  circumference (cm) | 95.50±10.33 | 94.18±11.72 | 95.59±9.05 | 97.69±8.94 |
| Hip  circumference (cm) | 99.73±6.48 | 99.56±7.38 | 100.23±8.06 | 100.42±5.30 |
| Waist-to-hip ratio | 0.95±0.06 | 0.94±0.07 | 0.96±0.05 | 0.97±0.06 |
| Smoking | 39(69.6%) | 37(66.1%) | 40(65.6%) | 44(72.1%) |
| Drinking | 43(76.8%) | 38(67.9%) | 39(63.9%) | 41(67.2%) |
| Hypertension | 25(44.6%) | 25(44.6%) | 28(45.9%) | 34(55.7%) |
| Fatty liver | 21(37.5%) | 24(42.9%) | 17(27.9%) | 31(50.8%) |
| Coronary heart  disease | 9(16.1%) | 11(19.6%) | 12(19.7%) | 20(32.8%) |
| Osteoporosis | 3(5.4%) | 4(7.1%) | 2(3.3%) | 4(6.6%) |
| History of cerebral  hemorrhage | 0(0.0%) | 0(0.0%) | 0(0.0%) | 2(3.3%) |
| History of cerebral  infarction | 5(8.9%) | 9(16.1%) | 11(18%) | 13(21.3%) |
| Diabetic peripheral neuropathy | 39(69.6%) | 38(67.9%) | 37(60.7%) | 45(73.8%) |
| Diabetic retinopathy | 16(28.6%) | 12(21.4%) | 16(26.2%) | 19(31.1%) |
| Heart rate | 74.00(72.00,78.00) | 72.00(71.75,78.00) | 74.00(71.00,78.00) | 76.00(72.00,78.00) |
| SBP (mmHg) | 126.64±13.83 | 131.02±16.27 | 130.48±18.04 | 133.64±16.16 |
| HDL-c (mmol/L) | 0.92(0.82,1.05) | 0.96(0.85,1.11) | 1.01(0.85,1.17)^a^ | 1.02(0.92,1.27) |
| LDL-c (mmol/L) | 2.46(2.19,3.12) | 2.27(1.78,2.81) | 2.05(1.67,2.91)^a^ | 2.16(1.67,2.50) |
| Total cholesterol  (mmol/L) | 4.29±0.92 | 3.94±0.95 | 3.98±0.89 | 3.79±0.94 |
| Triglyceride (mmol/L) | 1.58(1.04,2.32) | 1.50(1.08,2.55) | 1.18(0.91,2.39) | 1.25(0.87,1.91) |
| FPG (mmol/L) | 7.80(6.27,10.43) | 6.55(5.50,8.35) | 8.00(6.30,10.50) | 6.20(5.40,8.40) |
| HbA1c (%) | 9.30(7.90,10.20) | 8.20(7.25,9.35) | 9.00(7.20,11.03) | 8.50(7.10,9.67) |
| Uric acid (μmol/L) | 346.02±89.26 | 398.66±307.08 | 323.23±83.47 | 349.05±93.40 |
| AST (U/L) | 22.62±13.37 | 19.29±6.75 | 20.05±13.71 | 17.33±7.00 |
| Albumin (g/L) | 41.70±2.70 | 41.05±2.83 | 40.80±2.56 | 39.36±3.22^a^ |
| Urinary AER  (μg/min) | 19.11±17.04 | 36.09±53.70 | 57.16±146.13 | 24.72±36.76 |
| TSH (μIU/mL) | 1.83(1.34,3.03) | 1.83(1.38,2.86) | 1.81(1.18,2.70) | 2.09(1.34,3.13) |
| FT3 (pg/mL) | 2.94±0.43 | 2.97±0.39 | 3.01±0.34 | 3.02±0.28 |
| FT4 (ng/dL) | 1.21±0.18 | 1.18±0.17 | 1.19±0.20 | 1.21±0.19 |

**Supplementary Table 3**. Baseline and Follow-Up Characteristics of Type 2 Diabetes Patients and Diabetes Duration < 10 Years: No Progression vs. Progression to Impaired eGFR. Data are presented as mean ± SD, number (percentage), or median (interquartile range). eGFR, estimated glomerular filtration rate; BMI, body mass index; SBP, systolic blood pressure; HDL-c, high-density lipoprotein cholesterol; LDL-c, low density lipoprotein cholesterol; FPG, fasting glucose; AST, aspartate aminotransferase; AER, albumin excretion rate; TSH, thyroid-stimulating hormone; FT3, free triiodothyronine; FT4, free thyroxine. ^a^p < 0.05 vs no progression at same time point.

| Variable | No progression (n = 76) | | Progression (n = 40) | |
| --- | --- | --- | --- | --- |
|  | Baseline | Follow-up | Baseline | Follow-up |
| Female | ― | 37(48.7%) | ― | 10(25%)^a^ |
| Age (y) | 49.70±12.45 | 54.12±12.44 | 58.85±8.59^a^ | 63.62±8.77^a^ |
| Diabetes duration (y) | 5.00(2.00,8.00) | 9.00(6.75,12.25) | 6.50(4.00,9.00)^a^ | 10.00(8.75,14.00)^a^ |
| Follow-up time (y) | ― | 4.31(3.25,5.40) | ― | 4.19(3.45,5.92) |
| Weight (kg) | 71.00(63.75,80.00) | 74.00(64.50,81.25) | 68.00(60.00,74.25) | 70.05(60.00,80.00) |
| BMI (kg/m^2^) | 25.59(23.44,27.54) | 25.96(23.93,27.76) | 24.22(22.88,26.66) | 24.54(22.73,27.66) |
| Waist  circumference (cm) | 93.66±10.48 | 94.84±10.66 | 93.66±9.30 | 96.09±8.34 |
| Hip  circumference (cm) | 99.91±5.89 | 99.53±7.13 | 98.82±8.19 | 100.50±5.23 |
| Waist-to-hip ratio | 0.94±0.07 | 0.95±0.07 | 0.96±0.07 | 0.96±0.05 |
| Smoking | 33(43.4%) | 29(38.2%) | 20(50%) | 25(62.5%)^a^ |
| Drinking | 34(44.7%) | 29(38.2%) | 21(52.5%) | 22(55%) |
| Hypertension | 30(39.5%) | 34(44.7%) | 22(55%) | 26(65%)^a^ |
| Fatty liver | 22(28.9%) | 33(43.4%) | 10(25%) | 19(47.5%) |
| Coronary heart  disease | 9(11.8%) | 12(15.8%) | 4(10%) | 14(35%)^a^ |
| Osteoporosis | 5(6.6%) | 10(13.2%) | 2(5%) | 5(12.5%) |
| History of cerebral  hemorrhage | 0(0.0%) | 0(0.0%) | 0(0.0%) | 1(2.5%) |
| History of cerebral  infarction | 7(9.2%) | 8(10.5%) | 7(17.5%) | 12(30%)^a^ |
| Diabetic peripheral neuropathy | 48(63.2%) | 51(67.1%) | 24(60%) | 27(67.5%) |
| Diabetic retinopathy | 24(31.6%) | 24(31.6%) | 9(22.5%) | 9(22.5%) |
| Heart rate | 74.00(72.00,78.00) | 74.00(72.00,78.00) | 72.00(70.00,74.00)^a^ | 75.50(71.50,78.00) |
| SBP (mmHg) | 129.38±14.05 | 131.12±17.12 | 129.78±17.30 | 135.20±13.77 |
| HDL-c (mmol/L) | 1.03(0.84,1.19) | 1.04(0.88,1.24) | 1.02(0.90,1.16) | 1.04(0.93,1.27) |
| LDL-c (mmol/L) | 2.54(2.16,3.18) | 2.48(2.02,2.97) | 2.22(1.66,3.18) | 2.22(1.77,2.56)^a^ |
| Total cholesterol  (mmol/L) | 4.52±1.13 | 4.33±1.10 | 4.15±1.01 | 3.86±0.98^a^ |
| Triglyceride (mmol/L) | 1.49(1.01,2.44) | 1.50(1.08,2.38) | 1.42(0.91,2.40) | 1.28(0.97,2.06) |
| FPG (mmol/L) | 8.05(6.18,10.25) | 7.00(5.85,9.33) | 7.55(6.10,9.77) | 6.45(5.40,8.60) |
| HbA1c (%) | 9.35(7.88,10.60) | 8.70(7.20,9.90) | 9.25(6.90,10.90) | 7.90(6.85,9.45) |
| Uric acid (μmol/L) | 321.59±83.86 | 437.41±552.74 | 331.95±69.67 | 340.65±80.42 |
| AST (U/L) | 19.50(16.00,26.25) | 18.00(14.00,24.00) | 16.50(15.00,21.00) | 16.00(14.00,20.00) |
| Albumin (g/L) | 41.76±2.76 | 40.29±3.17 | 40.92±2.38 | 39.48±3.60 |
| Urinary AER  (μg/min) | 31.92±37.68 | 41.24±48.72 | 36.24±47.66 | 29.48±42.52 |
| TSH (μIU/mL) | 1.80(1.35,3.01) | 1.98(1.42,2.91) | 1.73(1.18,2.43) | 2.20(1.30,3.12) |
| FT3 (pg/mL) | 2.92±0.42 | 3.12±1.44 | 2.90±0.30 | 2.93±0.31 |
| FT4 (ng/dL) | 1.20±0.18 | 1.20±0.21 | 1.16±0.16 | 1.21±0.19 |

**Supplementary Table 4**. Baseline and Follow-Up Characteristics of Type 2 Diabetes Patients and a Diabetes Duration of 10–20 Years: No Progression vs. Progression to Impaired eGFR. Data are presented as mean ± SD, number (percentage), or median (interquartile range). eGFR, estimated glomerular filtration rate; BMI, body mass index; SBP, systolic blood pressure; HDL-c, high-density lipoprotein cholesterol; LDL-c, low density lipoprotein cholesterol; FPG, fasting glucose; AST, aspartate aminotransferase; AER, albumin excretion rate; TSH, thyroid-stimulating hormone; FT3, free triiodothyronine; FT4, free thyroxine. ^a^p < 0.05 vs no progression at same time point.

| Variable | No progression (n = 41) | | Progression (n = 37) | |
| --- | --- | --- | --- | --- |
|  | Baseline | Follow-up | Baseline | Follow-up |
| Female | ― | 27(65.9%) | ― | 15(40.5%)^a^ |
| Age (y) | 57.37±7.72 | 62.22±8.00 | 60.89±8.08 | 66.22±7.84^a^ |
| Diabetes duration (y) | 15.00(13.00,18.00) | 20.00(18.00,21.00) | 17.00(14.00,18.00) | 21.00(19.50,24.00) |
| Follow-up time (y) | ― | 4.60(3.71,6.23) | ― | 5.34(4.31,6.49) |
| Weight (kg) | 68.90(57.00,77.00) | 69.00(62.00,75.70) | 70.00(64.00,78.50) | 70.00(62.00,77.00) |
| BMI (kg/m^2^) | 24.26(22.32,27.28) | 24.82(21.95,26.88) | 25.41(23.31,27.55) | 24.77(23.24,26.26) |
| Waist  circumference (cm) | 92.50±10.25 | 99.71±7.67 | 95.38±9.33 | 94.11±8.75 |
| Hip  circumference (cm) | 97.33±7.55 | 102.71±7.97 | 100.08±7.53 | 99.11±5.93 |
| Waist-to-hip ratio | 0.94±0.05 | 0.97±0.06 | 0.95±0.06 | 0.95±0.08 |
| Smoking | 10(24.4%) | 11(26.8%) | 15(40.5%) | 15(40.5%) |
| Drinking | 11(26.8%) | 10(24.4%) | 12(32.4%) | 13(35.1%) |
| Hypertension | 9(47.4%) | 28(68.3%) | 21(56.8%) | 22(59.5%) |
| Fatty liver | 20(48.8%) | 19(46.3%) | 10(27%)^a^ | 16(43.2%) |
| Coronary heart  disease | 10(24.4%) | 14(34.1%) | 11(29.7%) | 14(37.8%) |
| Osteoporosis | 8(19.5%) | 8(19.5%) | 2(5.4%) | 4(10.8%) |
| History of cerebral  hemorrhage | 1(2.4%) | 1(2.4%) | 0(0.0%) | 1(2.7%) |
| History of cerebral  infarction | 6(14.6%) | 8(19.5%) | 7(18.9%) | 8(21.6%) |
| Diabetic peripheral neuropathy | 28(68.3%) | 27(65.9%) | 28(75.7%) | 28(75.7%) |
| Diabetic retinopathy | 10(24.4%) | 13(31.7%) | 9(24.3%) | 14(37.8%) |
| Heart rate | 74.00(72.00,78.00) | 74.00(70.00,79.00) | 76.00(72.00,78.00) | 74.00(70.00,78.00) |
| SBP (mmHg) | 130.83±15.49 | 135.12±17.86 | 130.51±17.08 | 131.08±17.78 |
| HDL-c (mmol/L) | 1.02(0.90,1.11) | 1.09(0.98,1.25) | 1.06(0.84,1.28) | 1.12(0.89,1.32) |
| LDL-c (mmol/L) | 2.42(1.89,3.12) | 1.73(1.38,2.35) | 2.36(1.76,3.06) | 2.21(1.74,2.53)^a^ |
| Total cholesterol  (mmol/L) | 4.10±0.99 | 3.50±0.89 | 4.19±0.97 | 4.08±1.08^a^ |
| Triglyceride (mmol/L) | 1.43(1.02,2.08) | 1.16(0.90,2.12) | 1.53(0.92,2.58) | 1.47(0.95,2.23) |
| FPG (mmol/L) | 7.50(5.90,8.70) | 7.20(5.70,8.80) | 7.30(6.40,10.50) | 7.00(6.20,8.90) |
| HbA1c (%) | 8.48±1.46 | 8.71±1.67 | 9.36±1.97^a^ | 9.38±1.79 |
| Uric acid (μmol/L) | 326.76±89.41 | 329.73±80.60 | 322.03±87.12 | 400.38±210.24 |
| AST (U/L) | 18.00(16.00,21.00) | 19.00(16.00,23.00) | 18.00(13.00,21.00) | 18.00(15.00,20.00) |
| Albumin (g/L) | 41.39±1.95 | 40.76±2.88 | 40.84±2.48 | 39.22±2.75^a^ |
| Urinary AER  (μg/min) | 33.33±33.69 | 39.25±48.71 | 81.34±174.99 | 26.21±31.44 |
| TSH (μIU/mL) | 1.87(1.67,3.30) | 1.63(1.00,2.84) | 1.83(1.09,3.32) | 2.08(1.03,2.92) |
| FT3 (pg/mL) | 3.09±0.38 | 3.04±0.37 | 3.20±0.38 | 2.94±0.45 |
| FT4 (ng/dL) | 1.16±0.17 | 1.18±0.18 | 1.26±0.22 | 1.24±0.22 |
